# Reduced ADAMTS13 activity is associated with an ADAMTS13 SNP, fever and microparticles in a malaria-like model

**DOI:** 10.1186/1475-2875-13-3

**Published:** 2014-01-03

**Authors:** Sirima Kraisin, Attakorn Palasuwan, Supaluk Popruk, Duangdao Nantakomol

**Affiliations:** 1 Department of Clinical Microscopy, Faculty of Allied Health Sciences, Chulalongkorn University, Bangkok, Thailand; 2 Department of Protozoology, Faculty of Tropical Medicine, Mahidol University, Bangkok, Thailand

**Keywords:** ADAMTS13, Cell-derived microparticles, Fever, *Plasmodium falciparum*, Malaria, Single nucleotide polymorphism

## Abstract

**Background:**

Severe falciparum malaria (SM) remains a major cause of death in tropical countries. The reduced activity of ADAMTS13, increasing levels of ultra-large von Willebrand factor (ULVWF) in SM patients, are assumed as factors that intensify disease severity. However, the reason why ADAMTS13 activity is reduced in SM remains unclear.

**Objectives:**

To investigate whether rs4962153, febrile temperature, and microparticles, contribute to reduced ADAMTS13 activity.

**Methods:**

Genotypic association of rs4962153 with ADAMTS13 antigen and activity was examined in 362 healthy Thai participants. The collagen binding assay was used to study the effects of febrile temperature and microparticles on ADAMTS13 activity.

**Results:**

ADAMTS13 antigen and activity were decreased in participants with AA genotype, compared to AG and GG (antigen: *p*-value = 0.014, and < 0.001; activity: *p*-value = 0.036, and < 0.002, respectively). There was significantly reduced ADAMTS13 antigen in AG compared to GG (*p*-value = 0.013), but not in ADAMTS13 activity (*p*-value = 0.082). The number of rs4962153 A alleles correlated with the reduced level of antigen and activity (*p*-value <0.001 and *p*-value = 0.001, respectively). MPs showed an inhibitory effect on ADAMTS13 activity (*p*-value = 0.025). Finally, ADAMTS13 activity was decreased in a temperature and time-dependent manner. The interaction between these two factors was also observed (*p*-value <0.001).

**Conclusions:**

These findings suggest that the A allele of rs4962153, MPs, and febrile temperature, contribute to reduce ADAMTS13 activity in plasma. These data are useful in malaria or other diseases with reduced ADAMTS13 activity.

## Background

*Plasmodium falciparum* malaria is a life-threatening disease in tropical countries, which causes approximately 500 million clinical cases, resulting in about 650,000 deaths each year [1]. Cerebral malaria (CM) is the most serious complication of severe malaria (SM) contributing to cause of death. Central pathophysiology of CM is the sequestration of parasitized red blood cells (PRBCs) in the cerebral microvasculature, which leads to microvascular obstruction and endothelial activation, including the release of von Willebrand factor (vWF) from its storage site in the endothelium, Weibel-Palade bodies [2]. However, the pathogenesis of CM remains poorly understood.

vWF is a large multimeric plasma glycoprotein that plays an important role in primary haemostasis by mediating the adhesion of platelets to sites of vascular injury. Normally, vWF and platelet-decorated vWF string is cleaved and regulated by an endogenous protease, a disintegrin and metalloproteinase with a thrombospondin type 1 motif, member 13 (ADAMTS13). Interestingly, the level of vWF and its propeptide are increased in SM patients compared to uncomplicated malaria and healthy control, and the increased level related with disease severity [3]. Moreover, there was abnormal ULVWF, a highly active form of vWF contributing to large platelet adhesion and aggregation to the vessel wall, in SM and CM patients rather than normal control [4,5]. These data correspond with the reduced activity of ADAMTS13 in SM and CM patients [4,6]. Furthermore, most symptoms of CM including fever, renal failure, microangiopathic haemolytic anaemia, neurological deficits and thrombocytopaenia, are commonly found in thrombotic thrombocytopaenic purpura (TTP), a rare life-threatening disease, which is caused by a congenital or acquired deficiency of ADAMTS13. Consequently, the factors causing the reduction of ADAMTS13 antigen and activity are, therefore, important in the pathogenesis of SM and CM.

It is well known that mutations in *ADAMTS13* gene contribute congenital TTP. A relatively large number of mutations and polymorphisms have been identified in *ADAMTS13*[7]. Recent study reported that the A allele of an intronic SNP, rs4962153, is associated with decreased risk of CM [8]. However, the effect of this SNP to expression level and activity of ADAMTS13 have not been studied.

A number of studies reported that level of MPs is increased in plasma with SM and CM, and correlated with disease severity [9-11]. MPs are vesicular particles released from blood cells, from which their functionalities are dependent on their original cell types. Surprisingly,MPs enhance the function of vWF during vascular injury, causing formation and more stability of platelet aggregation [12]. Although, some studies suggested that MPs derived from endothelial cells and platelets seem to have inhibitory capacity on ADAMTS13 activity [13,14], those are very preliminary studies. Activity of ADAMTS13 in plasma is quite stable. No significant decrease in ADAMTS13 activity is observed during storage at room temperature for up to 48 hr and 37°C for longer than one week; however, whether febrile temperature has any effect on ADAMTS13 activity has not been reported [15,16]. As fever is one of the symptoms established in both of TTP and SM, suggesting that effects of febrile temperature to ADAMTS13 activity should be investigated.

The aim of the present study was, therefore, to examine the effects from interesting factors that were found in SM on ADAMTS13. Genotypic association between rs4962153 and ADAMTS13 was explored in the Thai population. In addition, ADAMTS13 activity was measured in MPs-enriched plasma compared to MPs-depleted plasma. Finally, the activity of ADAMTS13 was determined in febrile-like plasma samples compared to normal plasma.

## Methods

### Subjects

A total of 362 healthy Thai people aged 18–44 years old were recruited in this study (254 females and 108 males). Those participants were healthy without fever, inflammation, infection, and taking of any anti-inflammatory medicines for at least seven days before blood collection. Furthermore, the participants had no medical history of any coagulopathies including haemophilia, thromboembolism, bleeding disorders, heart diseases, and severe inflammatory diseases. This study has approved by the Ethics Review Committee for Research Involving Human Research Subjects, Health Science Group, Chulalongkorn University, Thailand. Written informed consent was obtained from all participants.

### DNA extraction

Genomic DNA was extracted from EDTA blood using a FavorPrep™ FABGK genomic DNA Extraction mini kit according to the manufacturer’s instruction (Favorgen, Australia).

### Genotyping

rs4962153 (g.41635A > G), a significant SNP of *ADAMTS13* gene associated with cerebral malaria [8], was genotyped by TaqMan SNP Genotyping Assay for 362 healthy participants using StepOnePlus™ Real-Time PCR systems (Applied Biosystems, USA).

### Genotypic association study

34 of 362 participants were selected by convenience sampling and classified by their genotypes into three groups, which are GG, AG, or AA (19, 13 and two samples, respectively). Citrated plasma samples were collected from those participants and then the level of ADAMTS13 antigen and activity were performed using commercial kits (Quantikine Human ADAMTS13 ELISA kit; R&D Systems Inc., U.S.A, and Technozyme ADAMTS13 activity ELISA; Technoclone, Austria). The level of ADAMTS13 antigen and activity in each sample was determined in duplicate.

### Effect of microparticles on ADAMTS13 activity

Nineteen participants with genotype GG of rs496215. Three who presented the range of ADAMTS13 activity from 80 to 90% were enrolled in this study. The plasma samples were first prepared from platelet rich plasma (PRP), which was carefully collected after centrifugation at 250 *g* for 15 min at room temperature. The PRP was then centrifuged at 13,000 *g* for 3 min at room temperature. The MPs-enriched plasma was taken, which is referred to as ‘Non-filtered (NF)’ plasma throughout this article. The residual plasma was exhaustively filtered using Mimisart® Syringe filter hydrophilic with a pore size of 0.2 μm (Sartorius Stedim Biotech, Germany) to deplete MPs. The MPs-depleted plasma will be indicated throughout this article as ‘Filtered (F)’ plasma. Flow cytometry was used to confirm the events of MPs in both of NF and F plasma samples as described previously [11]. The ADAMTS13 activity was measured by the Collagen Binding Assay (CBA) as described below.

### Effect of febrile temperature on ADAMTS13 activity

The same citrated plasma samples from 30 participants as recruited in MPs test were used in this study. The aliquot samples were separately incubated at three different temperatures which are 37°C, 38°C and 39°C for 0, 3, 6, 12, 24 and 48 hr in each temperature. The ADAMTS13 activity was measured by the CBA.

### Measurement of ADAMTS13 activity by the collagen binding assay

To measure the effects of febrile temperature and MPs, the ADAMTS13 activity was analyzed in 30 citrated plasma samples by CBA, as described with slight modifications [17]. Importantly, febrile plasma samples which were previously incubated at 38°C and 39°C in plasma preparation process would be incubated at 38°C and 39°C instead of 37°C in digestion step, respectively. The chromogenic reaction in the final process was performed by addition of 100 μl of substrate o-phenylene-diamine dihydrochloride (5 mg) (Med OPD P6912, Sigma Aldrich, USA). The ELISA reaction was stopped by addition of 100 μl H_2_SO_4_ (3%) and absorbance was read at 492 nm with reference length at 620 nm using Sunrise™ absorbance reader (Tecan Group Ltd. Switzerland). The ADAMTS13 activity in each sample was determined in duplicate.

Normal pooled plasma (NPP), which was collected from 30 healthy participants was used to generate a calibration curve. The NPP was diluted in to 7 dilutions, 1:5, 1:10, 1:20, 1:40, 1:80, 1:160 and 1:320 in 1.5 M urea, 5 mM Tris, pH 8.0. The ADAMTS13 activity was read from the sigmoid-like calibration curve using point-to-point plot. The NPP dilution of 1:10 was defined as 107% of ADAMTS13 activity.

### Statistical analyses

Data are presented as means ± SEMs and analyzed by the software SPSS version 17.0 (SPSS Inc.). Statistical significances in genotypic association and effect of febrile condition were assessed by One-way ANOVA and Two-way ANOVA with repeated measures, respectively. A correction for multiple comparisons between individual groups was made using Tukey-HSD *Post-Hoc* analysis. Effect of MPs on ADAMTS13 was assessed using the paired *t*-test. Linear regression was used to describe the correlation between number of A allele of rs4962153 and ADAMTS13 antigen as well as activity as previously described [18]. Briefly, genotypes of all participants were coded as GG = 0, AG = 1, and AA = 2, according to the number of A allele. The *p*-value of less than 0.05 was considered to be of statistical significance.

## Results

### Frequency of rs4962153 SNP in Thai population

Among the 362 healthy Thai people, the frequencies of rs4962153 of *ADAMTS13* gene were 337 (93.10%) of genotype GG, 23 (6.35%) of genotype AG, and 2 (0.55%) of genotype AA. The minor allele frequency of this SNP was 3.98%, whereas heterozygote frequency was 6.35%.

### Genotypic association study

**Figure 1:**
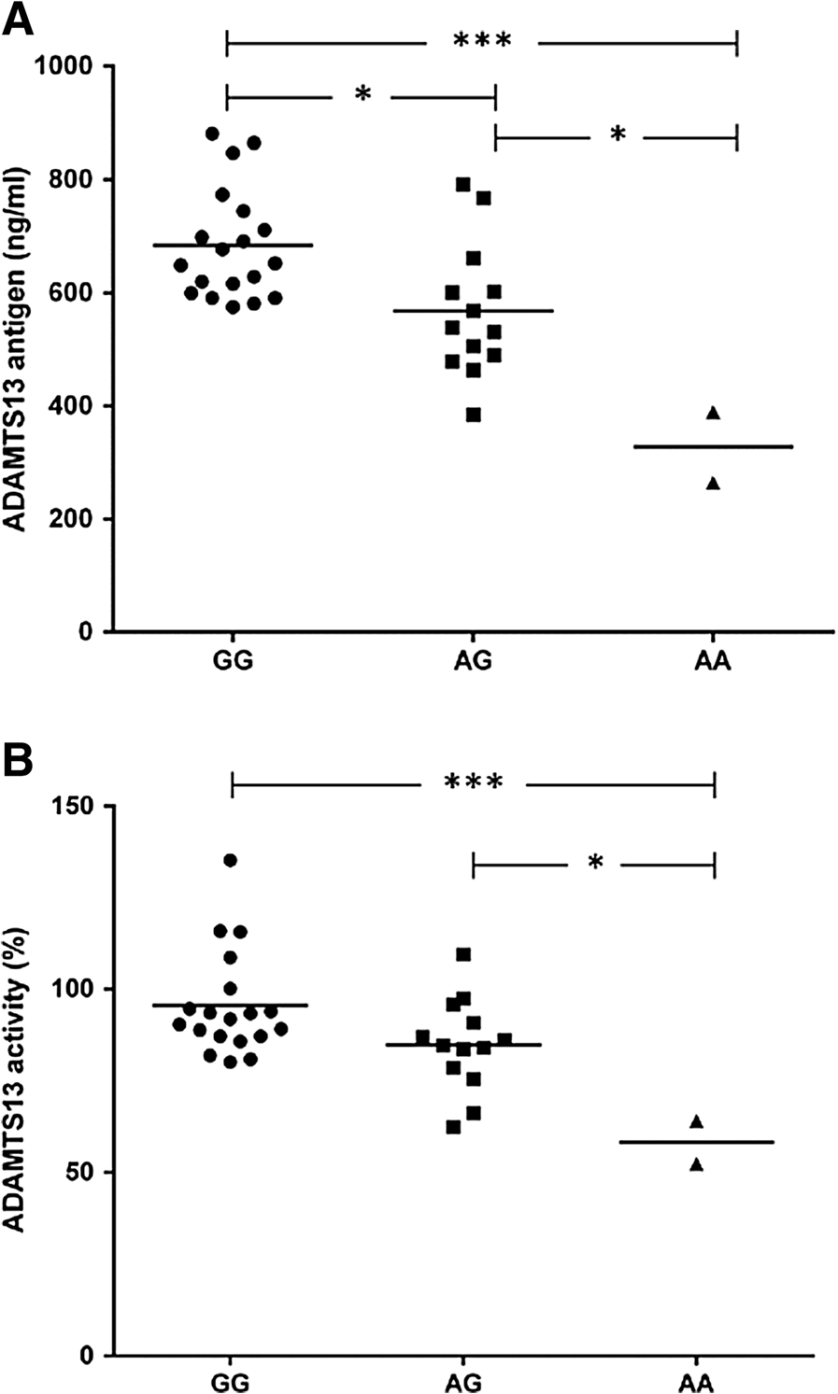
**Genotypic association of rs4962153 genotype and ADAMTS13 antigen and activity. (A)** The mean of ADAMTS13 antigen levels in participants with AA, AG and GG genotype of rs4962153 were 683.91, 568.02 and 327.26 ng/ml, respectively. ADAMTS13 antigen levels in participants with AA genotype were significantly reduced compared to both of AG and GG genotype (*P* < 0.05 and < 0.001, respectively). Furthermore, ADAMTS13 antigen in participants with AG genotype was also significantly decreased when compared to GG genotype (*P* < 0.05). **(B)** ADAMTS13 activity levels in participants with AA genotype (mean = 58.26%), were significantly reduced when compared to AG genotype (mean = 84.78%; *P* < 0.05) and GG genotype (mean = 95.53%; *P* < 0.001). The middle line referred mean of each individual genotype. *P < 0.05, **P < 0.01, ***P < 0.001.

ADAMTS13 antigens were remarkably decreased in participants with the AA genotypes of rs4962153 (mean ± SEM = 327.26 ± 62.53 ng/ml), compared to AG and GG genotypes (mean ± SEM = 568.02 ± 32.54 ng/ml, *p*-value = 0.014, and mean ± SEM = 683.91 ± 22.43 ng/ml, *p*-value < 0.001, respectively). Similarly, the participants with AA genotype represent the significant reduction of ADAMTS13 activity (mean ± SEM = 58.26 ± 5.83%), compared to the individuals with AG (mean ± SEM = 84.78 ± 3.5%, *p*-value = 0.036), and GG genotypes (mean ± SEM = 95.53 ± 3.25%, *p*-value = 0.002). It should be noted that the comparison of ADAMTS13 antigen between individuals with GG and AG was significantly different, whilst no significant difference of ADAMTS13 activity was observed between those individuals (*p*-value = 0.013 and 0.082, respectively; Figure 1).

### Effect of microparticles on ADAMTS13 activity

**Figure 2:**
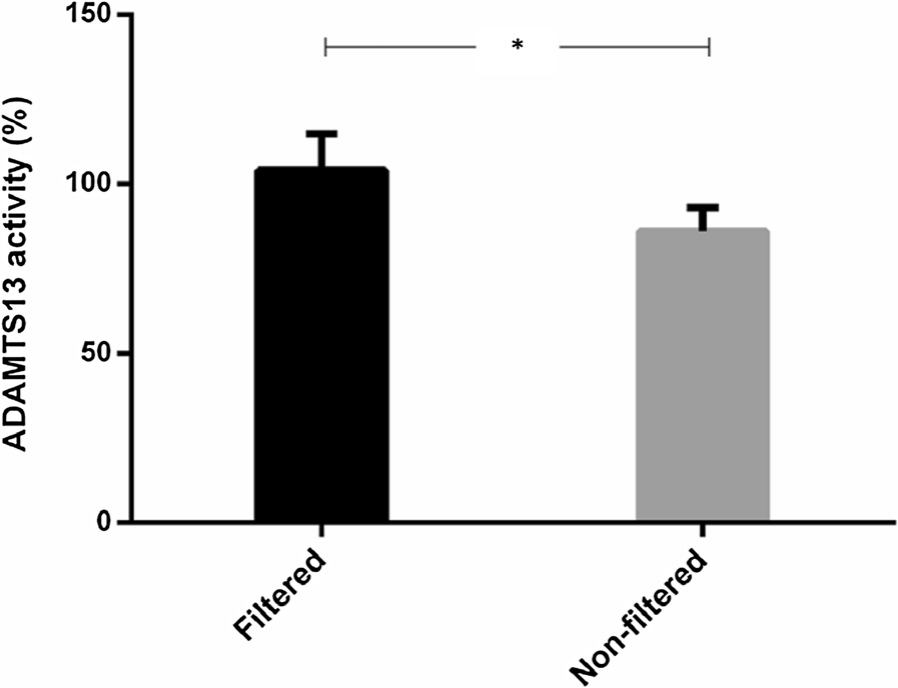
**Inhibitory effect of MPs on ADAMTS13 activity.** Non-filtered plasma samples presented ADAMTS13 activity 86.06%, which was significant lower than the filtered-plasma samples (mean = 104.03%). *P < 0.05.

The median (range) of MPs was significantly higher in NF plasma (2,546 (1198–3,432/μl)) than F plasma (5 (2-10/μl), *p-*value < 0.01). Interestingly, ADAMTS13 activity in NF plasma was significantly decreased (mean ± SEM = 86.06 ± 6.94%), when compared to F plasma (mean ± SEM = 104.03 ± 10.78%, *p*-value = 0.025; Figure 2). These data could support the inhibitory role of MPs on ADAMTS13 activity in plasma.

### Effect of febrile temperature on ADAMTS13 activity

**Figure 3:**
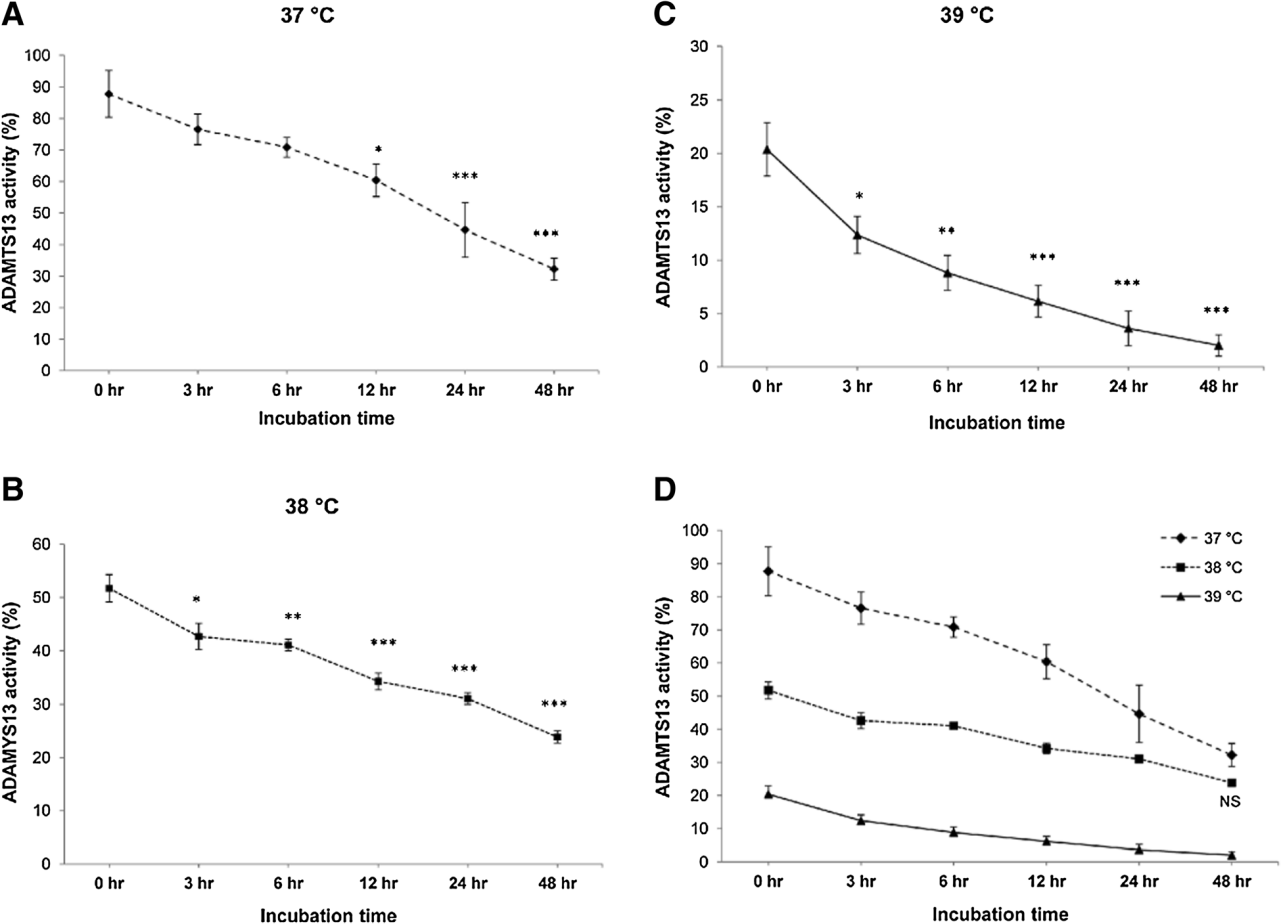
**ADAMTS13 activity was reduced in the incubation time and temperature-dependent manners. (A, B &C)** Clear evidence of a time-dependent inhitition was observed either at 37°C, 38°C, or 39°C. *P < 0.05, **P < 0.01, ***P < 0.001. **(D)** A temperature-dependent inhibition was observed. ADAMTS13 activity was significantly decreased at 38°C and 39°C incubation temperature, compared to 37°C for each incubation time except for 24 hr of 38°C compared to 37°C (NS = Not significant). All results represent mean ± SEM.

To investigate the effects from febrile temperatures, incubation times, including the interaction effect between temperatures and incubation time on ADAMTS13 activity, we used ANOVA with repeated measures (Table 1). ADAMTS13 activities were different for the 6 incubations times (*p*-value < 0.001). The significant differences in ADAMTS13 activity observed in plasma samples, incubated at 37°C, 38°C and 39°C (*p*-value < 0.001), indicate that temperature influences ADAMTS13 activity. Moreover, incubation time and temperature also showed the significant interaction effect on ADAMTS13 activity (*p*-value < 0.001). This effect means that the level of ADAMTS13 activities at different temperatures was different after incubation for 0 hr, 3 hr, 6 hr, 12 hr, 24 hr, and 48 hr. Indeed, following incubation at 37°C, 38°C and 39°C, clear reductions in ADAMTS13 activity were observed in a time-dependent manner (Figure 3). Following 6 hr of incubation at 37°C, there was no significant reduction in ADAMTS13 activity found when compared to 0 hr. However, after continuous incubation, the level of ADAMTS13 activity was significantly decreased at 12 hr, 24 hr, and 48 hr (Figure 3A). It should be noted that ADAMTS13 activity in normal plasma was reduced by approximately 50% after incubation at 37°C for 48 hr. Similarly, the ADAMTS13 activity was initially declined after incubation at 38°C for 3 hr, compared to 0 hr. More significant reduction corresponded with increased incubation time; the level of ADAMTS13 activity following incubation for 48 hr at 38°C was decreased compared to 0 hr, by approximately 30%. These data might indicate that ADAMTS13 activity is more stable in 38°C than 37°C after incubation for 48 hr (Figure 3B). Moreover, the incubation at 39°C represented the strongest reduction of ADAMTS13 activity, as the inhibitory effect was initially observed in plasma samples that were not incubated in preparation process (0 hr). The inhibitory effect remains after incubation for 3 hr to 48 hr in a time-dependent manner. Interestingly, ADAMTS13 was almost absent after the incubation for 48 hr under 39°C (mean ± SEM = 2.01 ± 1.01%; Figure 3C). These data led us to assume that the level of ADAMTS13 activity in plasma samples without incubation in the preparation process (0 hr) was declined by approximately 30% and 60% after incubated at 38°C and 39°C during the digesting process in CBA, respectively, compared to the same plasma incubated under normal conditions (37°C).

**Table 1:** Effects of Febrile temperatures and Incubation time to ADAMTS13 activity*

**Factors**	**df**	**F**	***p*-value**
Incubation time	5	40.086	<0.001
Temperature	2	138.35	<0.001
Incubation time * Temperature	10	8.063	<0.001

*Incubation time *temperature = interaction effect of temperature and incubation time; df = degree of freedom; F = F-test score.

Clear evidence of the temperature-dependent inhibitory effect was observed (Figure 3D). ADAMTS13 activities were most inhibited at 39°C, and the least inhibitory effect observed at 37°C, with moderate inhibition at 38°C. To confirm these events, *Post-Hoc* analysis was used to assess the differences of ADAMTS13 activity at all temperatures, 37°C versus 38°C, 37°C versus 39°C, and 38°C versus 39°C, in each incubation time. The result showed that there were significant differences observed between ADAMTS13 activities in most comparisons at each incubation time except the comparison between 37°C and 38°C at 24 hr (*p*-value = 0.187; Table 2).

**Table 2:** Level of ADAMTS13 activity in plasma incubated in normal and febrile temperature

**Incubation time (hr.)**	% ADAMTS13 activity (mean ± SEM*)			***p*-value (*Post-Hoc *** **Tukey HSD)**		
	**37°C**	**38°C**	**39°C**	**37°C vs 38°C**	**37°C vs 39°C**	**38°C vs 39°C**
0	87.75 ± 7.39	51.73 ± 2.57	20.36 ± 2.49	*<* 0.001	< 0.001	0.001
3	76.57 ± 4.89	42.67 ± 2.45	12.36 ± 1.73	< 0.001	< 0.001	< 0.001
6	70.87 ± 3.11	41.08 ± 1.09	8.80 ± 1.64	< 0.001	< 0.001	< 0.001
12	60.38 ± 5.19	34.24 ± 1.60	6.14 ± 1.49	< 0.001	< 0.001	< 0.001
24	44.65 ± 8.65	31.04 ± 1.03	3.60 ± 1.62	0.187	< 0.001	0.007
48	32.22 ± 3.49	23.83 ± 1.17	2.01 ± 1.01	0.048	< 0.001	< 0.001

*SEM = Standard Error of the Mean.

## Discussion

A number of previous studies have shown that the level of ULVWF and vWF multimers are higher in patients with SM and CM, together with decreased antigen and activity of ADAMTS13, compared to healthy controls [4,6]. The present finding revealed that AA genotype of rs4962153 associated with notably reduced activity and antigen of ADAMTS13 in healthy participants, when compared to AG and GG genotypes. The number of A allele showed strong association with reduced ADAMTS13 antigen and activity, which could confirm allele A is significant. Moreover, MPs have inhibitory effects on ADAMTS13 activity in normal plasma. Finally, febrile temperature and incubation time themselves and interaction together significantly decreased ADAMTS13 activity in temperature and time-dependent manner.

In recent years genetic variation in *ADAMTS13* gene associated with congenital TTP has been reported [7]. Interestingly, a previous study has shown that an intronic SNP, rs4962153-A, was significantly associated with protection against CM in Thai patients [8]. This study was designed to investigate the genotype and allele frequency in 362 healthy Thai participants. Considering genotype frequency, AA genotype was rarely found in those participants. Correspond with minor allele frequency (A allele), which was less than 5% in this study. HapMap project revealed that this SNP is unusually found in Asian population, including Chinese, and Japanese (5.8%, and 9.9%, respectively), whereas, commonly found in European and sub-Saharan African (15.6% and 38.5%, respectively) [19]. In this recent study, it was found that AA genotype associated with reduced ADAMTS13 antigen and activity compared to AG and GG genotypes (Figure 1). Moreover, the significant difference of ADAMTS13 antigen was also noticed in a comparison between AG and GG genotype, but not in ADAMTS13 activity. Although only two participants with AA genotype were enrolled in this study, the individuals with GG genotypes had about two times higher in both ADAMTS13 antigen and activity (2.09 and 1.64, respectively). Furthermore, the level of antigen in participants with AA genotype (327.26 ± 62.53 ng/ml) was slightly lower than the minimal level of normal range for ADAMTS13 in citrate plasma (370–1403 ng/ml; Quantikine® ELISA human ADAMTS13 manual), the activity in those participants (58.26 ± 5.83%) was included in reference range (40–130%; TECHNOZYM® ADAMTS-13 Activity manual). These data may explain that even if the antigen level is low, there are some compensatory mechanisms that can support normal ADAMTS13 activity. However, a study in a larger Thai population or in other populations should be examined.

The A allele of rs4962153 was assumed to be a risk allele as it associated with the decreased ADAMTS13 antigen and activity in this present study. This finding supports a Swedish study, which reported that rs4962153-A was associated with susceptibility to ischemic stroke (IS) [20]. Therefore, rs4962153-A may reduce the ADAMTS13 activity in IS patients. On the other hand, the A allele was associated with decreased risk of CM in the previous study [8]. As the transcription level of *ADAMTS13* involved with rs4962153-A is unlikely increased or decreased depending on the situation, the reported associations may be false positive in either study. However, this present study examined in healthy participants *in vitro*, functional factors found in malaria patients may cause the difference. To assess the results, the effect of this SNP on mRNA and/or ADAMTS13 antigen as well as its activity in malaria patients should be further investigated. The information about rs4962153 is not much investigated. It is an intronic SNP without known function. This SNP is possibly in strong linkage disequilibrium (LD) with a variant that regulates gene expression, or with a non-synonymous coding SNP. Moreover, alternative splice site spanning the location of rs4962153, including effect an enhancer/silencer encoded within the intron, should be considered.

Platelets and ECs are activated, and then apoptosis is increased in CM, which leads to MPs release in those cells. MPs are submicronic membranous rudiments characterized by the elevated exposure on the external membrane leaflet of anionic phospholipids, such as phosphatidylserine (PS), carrying on their surface proteins from the original cell types, which give them specific biological properties [21]. Platelets are able to modulate PRBC cytoadherence and PMPs represent the majority of circulating MPs. PMPs transfer platelet antigens such as PECAM-1 and CD36, which are major receptors supporting PRBC adhesion [10,22]. An initial process of thrombus formation via vWF-platelet interaction is the binding of platelet receptor GPIbα to the vWF A1 domain, resulting in the exposure of the binding and cleavage site for ADAMTS13 under shear stress [10]. The present study has found an inhibitory effect from MPs (mostly released from platelets) on ADAMTS13 activity. This finding agrees with a preliminary study, which implied that activated platelets and PMPs could regulate ADAMTS13 activity [14]. Altogether, the increased PMPs in circulation could possibly compete in binding ADAMTS13 by reducing and/or blocking its interaction with vWF, leading to conformational change and less multimeric cleavage. It seems unlikely PMPs alone strongly decrease ADAMTS13 activity as the activity in PMPs-enriched plasma remained in normal range; however, high level of PMPs together with other risk possibly contribute sturdily decrease ADAMTS13 activity and then increase risk of thrombosis.

The present data show that following incubation at 37°C for 48 hr, ADAMTS13 activity was slowly decreased, represent half-life about 24–48 hr *in-vitro*. The half-life is slightly shorter than the respective half-live (48–72 hr) observed in TTP patients [23]. This data is slightly in conflict with an *in-vitro* study which reported that ADAMTS13 was quite stable with half-life longer than 1 week [16]. Since the studies have been examined in different populations and under different conditions, further independent studies are needed to examine whether the findings can be confirmed. ADAMTS13 activity was reduced continuously during incubation with half-life spanning about 48 hr and 3 hr after incubation at 38°C and 39°C, respectively. Moreover the protease activity was markedly inactivated when the temperature was increased (Figure 3). It seems likely that ADAMTS13 activity is inactivated depending on time and temperature. In falciparum malaria, core temperature of patients may rise as high as 42°C. An *in-vitro* study revealed that fever accelerates and increases the cytoadherence of PRBCs to the major parasitized receptors, CD36 and ICAM-1 [24]. The possible mechanism may be related with the inactivated ADAMTS13 activitiy, which results insufficiently regulated the platelet-decorated ULVWF strings.

These findings show that ADAMTS13 activity is reduced in healthy Thai participants with AA genotype compared to AG and GG genotype, and seems related to a number of A alleles. Moreover, MPs and febrile temperature have the capacity to inhibit and/or inactivate ADAMTS13. The present findings are not implied only in malaria infection, but also in other diseases for example, TTP, heart disease, coagulopathies, and other inflammatory diseases.

## Competing interests

The authors declare that they have no competing interests.

## Authors’ contributions

SK drafted the manuscript and performed experiments. AP performed data analysis and interpretation. SP collected the samples. DN involved in providing the conception, design of the study and revised the manuscript critically for intellectual content and approved the final version of the manuscript. All authors read and approved the final manuscript.
